# Follow-up evaluation of pulmonary function and computed tomography findings in chronic kidney disease patients after COVID-19 infection

**DOI:** 10.1371/journal.pone.0286832

**Published:** 2023-08-15

**Authors:** Solos Jaturapisanukul, Nadwipa Yuangtrakul, Dearada Wangcharoenrung, Krongkan Kanchanarat, Kan Radeesri, Jakravoot Maneerit, Anan Manomaipiboon, Khemika Rojtangkom, Chompoonuth Ananthanalapa, Siwaporn Rungrojthanakit, Peerawit Thinpangnga, Joshua Alvior, Thananda Trakarnvanich

**Affiliations:** 1 Faculty of Medicine, Vajira Hospital, Navamindradhiraj University, Bangkok, Thailand; 2 College of Nursing and Health, Suan Sunandha Rajabhat University, Bangkok, Thailand; 3 USF Health Morsani College of Medicine, Tampa, Florida, United States of America; Aga Khan University, PAKISTAN

## Abstract

Pulmonary complications are common after SARS-CoV2- infection. However, data on pulmonary sequelae of COVID-19 after recovery in dialysis patients are limited. We determined the prevalence of abnormal lung function tests and CT findings and investigate the association factors impacting pulmonary dysfunction. This prospective observational cohort study enrolled 100 patients with stage 5 chronic kidney disease (CKD) undergoing dialysis who had recovered from COVID-19 for ≥3 months. Pulmonary function test (PFT) and chest computed tomography (CT) were performed. Demographic data and laboratory results were recorded. The mean patient age was 55.15 ± 12.84 years. Twenty-one patients (21%) had severe COVID-19, requiring mechanical ventilation or oxygen supplementation. Pulmonary function tests revealed a restrictive pattern in 41% (95% confidence interval [CI], 31.73–50.78;) and an obstructive pattern in 7.29% (95% CI, 3.19–13.25) patients. The severe group showed PFT test results similar to the non-severe group, with three patients showing severe obstructive lung disease. The CT scan findings included reticulation (64%), multifocal parenchymal band (43%), ground glass opacities (32%), and bronchiectasis (28%). The median total CT score was 3 (interquartile range, 1–8.5). The CT score and PFT findings showed no association with pulmonary dysfunction extent, except in bronchiectasis. Lung function indices were associated with abnormal CT findings. Abnormal CT findings (bronchiectasis, reticulation, and ground-glass opacities) was associated with higher oxygen requirements than normal CT findings (p = 0.008, bronchiectasis; p = 0.041, reticulation; p = 0.032, ground-glass appearance). Aside from CT findings and CRP levels, no significant lung abnormalities were observed in severe and non-severe patients. Some patients had residual symptoms at follow-up. The findings indicate persistence of both radiological and physiological abnormalities in dialysis patients after COVID-19. However, the prevalence of these abnormalities was comparable to that in the normal population; few patients experienced ongoing symptoms. Follow-up observations and evaluations are warranted.

**Trial registration. Clinicaltrials.gov Identifier**: NCT05348759.

## Introduction

Coronavirus disease 2019 (COVID-19), which is caused by severe acute respiratory syndrome-coronavirus 2 (SARS-CoV-2), is associated with increased morbidity and mortality in patients with chronic kidney disease (CKD) on dialysis [1,2]. CKD patients were particularly susceptible during the pandemic due to an increased exposure from frequent health care center visits for maintenance hemodialysis [3]. COVID-19 involved multiple organs, and lung injury was one of the most common clinical manifestations [4]. The binding of SARS-CoV-2 to the ACE2 receptors at target cells, including type II pneumocytes and alveolar macrophages in the lung, can induce acute systemic inflammatory responses and cytokine storms [5], leading to lung-resident dendritic cell (rDC) activation, T lymphocyte production, and release of antiviral cytokines into the alveolar septa and interstitial compartments and diffuse alveolar epithelium destruction, hyaline membrane formation, alveolar septal fibrous proliferation, and pulmonary fibrosis [6–8]. Although subgroups of COVID-19 survivors may show persistent lung parenchymal injury for at least 6 months [9,10], the long-term outcomes of COVID-19 as well as pulmonary function testing (PFT) results after COVID-19 in a population of CKD patients have not been investigated. Therefore, we aimed to assess the pulmonary sequalae and PFT results 3 months after recovery from COVID-19 in CKD patients undergoing hemodialysis (HD).

## Materials and methods

### Patient population

This prospective, observational cohort study was conducted in patients with CKD Stage 5 on renal replacement therapy (RRT), consisting of either HD or continuous ambulatory peritoneal dialysis (CAPD) who survived acute COVID-19, diagnosed using real-time polymerase chain reaction (RT-PCR) and presented for clinical follow-up at least 3 months after mild, moderate, or severe infection. The inclusion criteria were age between 18 and 80 years and CKD Stage 5 requiring RRT for at least 3 months. We excluded patients with pre-existing concomitant lung diseases such as chronic obstructive pulmonary disease (COPD), restrictive lung disease, asthma, or interstitial lung disease. The diagnostic criteria for severe COVID-19 were as follows: (1) oxygen saturation < 90% on room air, (2) severe pneumonia, (3) signs of severe respiratory distress (accessory muscle use, inability to complete full sentences, and respiratory rate > 30 breaths per minute), and (4) SpO_2_ < 93% at rest and PaO_2_/FiO_2_ < 300 mmHg. Non-severe COVID-19 was defined by the absence of any criteria for severe or critical COVID-19 [11,12]. The study was conducted at the Faculty of Medicine, Vajira Hospital, Navamindradhiraj University from June 2022 to January 2023. The study protocol adhered to the Declaration of Helsinki, and all patients provided written informed consent before enrolling in the study. Ethics approval was obtained from Institutional Review Board (IRB) of Faculty of Medicine,Vajira Hospital,Navamindradhiraj University prior to start of the study (COA No 302/64 E).

### Assessment of clinical data

We collected demographic data and information regarding disease history, coexisting medical conditions, medication history, treatment during COVID-19 infection, including oxygen requirement, and laboratory data (complete blood count [CBC] and measurement of interleukin-6 [IL-6] and C-reactive protein [CRP] levels). At least 3 months after COVID-19 infection, all patients were evaluated for ongoing respiratory symptoms and underwent PFT and chest CT scans as follows.

### Pulmonary function testing

Spirometry was performed by a trained nurse at the Division of Pulmonology, Vajira Hospital. The forced vital capacity (FVC), forced expiratory volume in the first second of exhalation (FEV1), forced mid-expiratory flow (FEF25%-75%), and the FEV1/FVC ratio before and after bronchodilator administration (2 puffs of salbutamol via spacer,100 μg each puff) were recorded in all cases. Measurements of total lung capacity (TLC) using spirometry (Masterscreen PFT; Jaeger, Germany) and the diffusion capacity of carbon monoxide (DLCO) were performed in selected cases due to lung parenchymal abnormalities detected in chest CT. PFT findings were interpreted in accordance with the global lung function 2012 equations [13]. as a reference standard. Obstruction is determined by FEV1/FVC ratio below lower limit of normal (LLN). Restriction is determined by FVC below LLN. Small airway disease is determined by FEV25-75% below LLN. Since, GIL2012 is a complicated equation and not being widely used in Thailand at the time of current study, the GLI2012 lower limit of normal was obtained using application “Spiro Thai version 3.0/4.0” (Supplementary Appendix 1) [14]. The Z-score was not calculated by the application at that time.

### Diagnostic test: Chest computed tomography scans

#### Computed tomography technique

High-resolution computed tomography (HRCT) was performed in a single breath-hold on a 128-slice multidetector computed tomography (MDCT) scanner (Ingenuity 128; Philips Healthcare Nederland B.V, Netherlands). HRCT was performed with a 1-mm slice thickness with the patient in the supine position during end-inspiration and prone position during end-inspiration.

#### Computed tomography interpretation

Using a Picture Archiving and Communication System (PACS; EV Insite version 3.11.1.500; PSP Corporation, Japan), three radiologists with 9, 10, and 14 years of experience performed consensus interpretations blinded to the patients’ clinical information. The evaluators assessed the presence of the following CT patterns; consolidation, ground-glass opacities (focal, multifocal, diffuse), mosaic attenuation patterns (hypoattenuating and hyperattenuating areas), perilobular consolidation (organizing pneumonia-like pattern), reticulations, architectural distortion, honeycombing, traction bronchiectasis, pneumatocele, curvilinear lines, nodules, and pleural thickening or pleural effusion [15]. Additional findings were annotated separately. The distribution of the patterns was recorded as the upper lobe, middle lobe/lingual, or lower lobe. CT scores reflecting the extent of lobar involvement were obtained using a five-point scale (0: 0%, 1: <5%, 2: 5%-25%, 3: 26%-50%, 4: 51%-75%, 5: >75%; range, 0–5; global score, 0–25) [16].

### Outcome measures

The primary outcomes were chest CT findings and PFT results in CKD stage 5 patients after recovery from COVID-19. Secondary outcomes included hospitalization-related factors affecting pulmonary sequalae after COVID-19 infection in CKD patients, such as oxygen requirement, ventilator requirement, and levels of laboratory markers (such as IL-6 and CRP).

### Sample size calculation

This study aimed to identify the prevalence of pulmonary abnormalities in both radiographic findings and PFT results after recovery from COVID-19 infection. We used the following equation for estimating an infinite population proportion [17]:

$$n_{}=\frac{{Z_{\alpha /2}}^{2}p(1-p)}{d^{2}}$$

where, *n* is the sample size

*Z*_*α/2*_ is the area under the normal curve

The significance level for the hypothesis was set to α = 0.05; thus Z_α/2_ = 1.96

d is the acceptable error (d = 0.10)

*p* is the prevalence of lung abnormality, defined as *p* = 0.50, that yielded the maximum sample size; thus,

$$n=\frac{1.96^{2}\;x\;0.50\;(1‐0.50)}{0.10^{2}}$$

n=97

We recruited 100 cases by convenience sampling from a population of end-stage kidney disease (ESKD) patients who had recovered from COVID-19 infection.

### Statistical analysis

Continuous variables were reported as mean and standard deviation or median and interquartile range (IQR), as appropriate. The PFT and CT scan results were reported as absolute and relative frequencies and percentages [%] of prevalence with the 95% confidence interval [95% CI]). The associations of various factors, such as ventilator usage, oxygen requirements, laboratory markers (IL-6 and CRP), mode of RRT, and changes in PFT and CT scan results were estimated using chi-squared test or Fisher’s exact test when the data were qualitative, and Kruskal–Wallis or Mann–Whitney U test when the data were quantitative. IBM SPSS Statistics for Windows, Version 26.0 (Armonk, NY, USA: IBM Corp) was used for all statistical analyses. Statistical significance was defined as p_(two-sided)_ ≤ .05.

## Results

### Participant characteristics

Between May and December 2022, a total of 100 patients (51 male and 49 female) who recovered from COVID-19 were enrolled in the study. Twenty-nine had been discharged from isolation wards while nine had been discharged from a “hospitel” (a hotel that was used as an isolation area for COVID-19 patients). The remaining 83 patients (83%) were isolated at home. All patients were followed up at least three months (median, 4 months; range, 3–9 months) after recovery from COVID-19. The mean patient age was 55.15 ± 12.84 years (range, 24–78 years). Baseline characteristics, laboratory findings, and pre-existing comorbidities are described in Table 1. The most common cause of CKD was diabetes mellitus (DM; 46%). Two patients (2%) were current smokers, one had a medical history of cancer, and 17 (17%) had heart diseases, (13 (13%) with ischemic heart disease and 4 (4%) with atrial fibrillation). Twenty-one (21%) patients had severe disease and needed intensive care, with 2 (2%) requiring mechanical ventilation. While 71 (71%) patients were undergoing HD, 29 (29%) were receiving CAPD. The median dialysis vintage time was 5 years (range, 3–8 years). Five patients (5%) were taking oral anticoagulation therapy due to atrial fibrillation before contracting COVID-19, and 31 patients (31%) were receiving antiplatelet agents due to a medical history of ischemic stroke. The remaining patients were discharged without anticoagulants. Seven patients (7%) were currently taking immunosuppressive drugs for underlying glomerular diseases.

**Table 1:** Baseline characteristics, treatment and laboratory data of 100 patients.

	n	%
**Sex**		
M	51	51.00
F	49	49.00
**Age (yr), mean ± SD**	100	55.15 ± 12.84
**Weight (kg), mean ± SD**	100	61.02 ± 11.58
**Height (cm), mean ± SD**	100	162.47 ± 10.37
**BMI (kg/m** ^**2**^ **), mean ± SD**	100	23.14 ± 4.06
**CKD_DM**		
N	61	61.00
Y	39	39.00
**CKD_HT**		
N	65	65.00
Y	35	35.00
**CKD_glomerulus**		
N	98	98.00
Y	2	2.00
**CKD_obstruction**		
N	100	100.00
Y	0	0.00
**CKD_other**		
N	78	78.00
Y	22	22.00
**CKD_unknown**		
N	83	83.00
Y	17	17.00
**Renal replacement therapy**		
HD	89	89.00
PD	11	11.00
**Dialysis vintage, median (IQR)**	100	5 (3–8)
**Post-covid duration, median (IQR)**	100	4 (3–9)
**Duration of admission, median (IQR)**	99	10 (7–13)
**ICU**		
N	90	90.00
Y	10	10.00
**Isolation ward**		
N	28	28.00
Y	72	72.00
**Hospital**		
N	91	91.00
Y	9	9.00
**HI**		
N	83	83.00
Y	17	17.00
**DM**		
N	54	54.00
Y	46	46.00
**HT**		
N	15	15.00
Y	85	85.00
**IHD**		
N	87	87.00
Y	13	13.00
**HF**		
N	100	100.00
Y	0	0.00
**AF**		
N	96	96.00
Y	4	4.00
**HIV infection**		
N	100	100.00
Y	0	0.00
**Cancer**		
N	99	99.00
Y	1	1.00
**Current smoking**		
N	98	98.00
Y	2	2.00
**ACEI/ARB**		
N	78	79.59
Y	20	20.41
**Antiplatelet drugs**		
N	67	68.37
Y	31	31.63
**Oral anticoagulants**		
N	94	94.95
Y	5	5.05
**Immunosuppressive drug**		
N	92	92.93
Y	7	7.07
**Number of vaccine doses**		
1	7	7.00
2	43	43.00
3	44	44.00
4	6	6.00
**Number of vaccines, median (IQR)**	100	2.5 (2–3)
**mRNA**		
N	47	47.00
Y	53	53.00
**Viral vector**		
N	15	15.00
Y	85	85.00
**Whole virus**		
N	87	87.00
Y	13	13.00
**Favipiravir**		
N	12	12.00
Y	88	88.00
**Andrographis**		
N	97	97.00
Y	3	3.00
**Remdisivir**		
N	91	91.92
Y	8	8.08
**Corticosteroid**		
N	91	91.00
Y	9	9.00
**Tocilizumab**		
N	100	100.00
Y	0	0.00
**Baciritinib**		
N	100	100.00
Y	0	0.00
**Hemoperfusion**		
N	100	100.00
Y	0	0.00
**IV anticoagulation**		
N	99	99.00
Y	1	1.00
**Ivermactin**		
N	97	97.00
Y	3	3.00
**Molnupiravir**		
N	99	99.00
Y	1	1.00
**Mechanical ventilation**		
N	96	96.00
Y	4	4.00
**O**_**2**_ **support**		
N	81	81.00
Y	19	19.00
**Cough**		
N	94	94.00
Y	6	6.00
**Dyspnea**		
N	86	86.00
Y	14	14.00
**Other symptoms**		
N	99	99.00
Y	1	1.00
**Hb (g/dL), mean ± SD**	97	9.71 ± 1.74
**WBC(10** ^**9**^ **/L), median (IQR)**	97	6.36 (5.2–8.9)
**PMN(%) (neutrophils), mean ± SD**	97	69.22 ± 12.35
**LYM(%), median (IQR)**	97	18.9 (12.9–22.7)
**Eosinophils (%), median (IQR)**	97	2.9 (0.9–6)
**Platelets (10** ^**9**^ **/L), median (IQR)**	97	205 (148–240)
**CRP (mg/L), median (IQR)**	63	12 (4.3–34.4)
**IL-6 (pg/mL), median (IQR)**	12	22.9 (8.32–29.35)
**Albumin (mg/dL), median (IQR)**	85	3.8 (3.6–4)
**Ferritin(ng/mL), median (IQR)**	10	777.5 (309–976)
**D-dimer (mg/L), median (IQR)**	21	1.1 (0.69–1.65)

ACEI, angiotensin converting enzyme inhibitor; AF, atrial fibrillation; ARB, angiotensin receptor blocker; CRP, C-reactive protein; CKD, chronic kidney disease; DM, diabetes mellitus; Hb, hemoglobin; HD, hemodialysis; HF, heart failure; HIV, human immunodeficiency virus; HT, hypertension; ICU, intensive care unit; IHD, ischemic heart disease; IL-6, interleukin-6; IQR, interquartile range; LYM, lymphocytes; PD, peritoneal dialysis; PMN, polymorphonuclear neutrophils; WBC, white blood cells

### COVID-19 treatment and laboratory data

For patients who required admission to either an isolation ward or the “hospital”, the median (IQR) admission duration was 10 days (7–13). While 53 patients received mRNA vaccines (53%), 85 received a viral-vector vaccine (ChAdOx1 nCoV-19), and eight received the inactivated vaccine. Nearly half (45%) of the total study population had received three doses of the COVID-19 vaccine, while eight patients had not received any vaccinations. Ninety-five patients had mild-to-moderate COVID-19, and 88 patients were treated with favipiravir, which was the main drug of choice at the time of study. None of the patients required hemoperfusion or treatments with antibodies such as tocilizumab, remdesivir, or baciritnib. However, nine patients required corticosteroid treatment. No correlation was observed between disease severity and vaccination history. The two patients requiring mechanical ventilation were placed in the severe group, while 19 other patients required oxygen support ranging from 3–10 L/min of which only one needed oxygen for more than 7 days.

Blood tests performed at admission showed normal D-dimer levels (median, 1.1 mg/L [0.69–1.65 mg/L]). The median CRP and IL-6 levels were 12 mg/dL (range, 4.3–34.4 mg/dL) and 22.9 pg/mL (range, 8.32–35.4 pg/mL), respectively. The CBC and albumin measurements were mostly in the normal range.

### Follow-up pulmonary function test after 3 months post COVID-19 infection (Fig 1)

**Fig 1:**
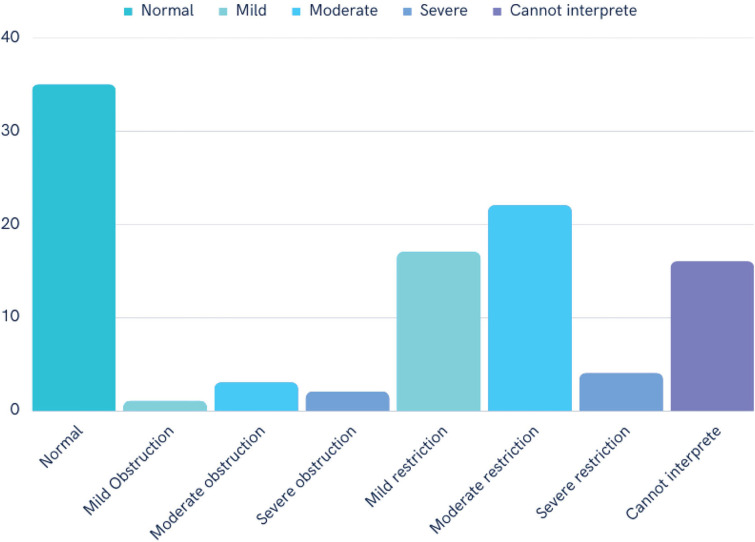
Follow-up pulmonary function test three months post COVID-19 recovery.

We categorized the PFT findings as normal, restrictive, obstructive, and “unable to interpret.” After excluding the missing data and tests that did not meet the acceptability criteria [18], there was 76 spirometry tests that could be interpreted. There were 30 normal spirometry defined by FVC% predicted ≥80% and FEV1/FVC > 0.7. Restrictive pulmonary dysfunction, as demonstrated by reduced FVC and/or TLC, was the most frequent post-COVID-19 lung abnormality in our cohort. A restrictive pattern was observed in 41% of the patients (95% CI, 31.73–50.78) of which 14 cases were mildly severe, 25 cases were moderate and 1 case had severe restrictive pattern. An obstructive pattern was observed in 7.29% (95% CI, 3.19–13.25) of which in two cases had mild severity, four cases had moderate and one case had severe obstruction. There were five cases of small airway disease, as determined by FEF_25-75_ being less than 65% of the predicted value. The “cannot interpret” category included cases showing an incomplete graph in PFT. Among the four severe cases (requiring mechanical ventilation), one showed mild restrictive lung dysfunction at the follow-up study, two showed moderate obstruction, and one was classified as “unable to interpret.” Among the 19 patients requiring oxygen therapy, three (14.27%) showed an obstructive pattern, four (19.05%) and seven (33.33%) were classified as “cannot interpret.” The remaining seven patients (33.33%) had a normal PFT.

### Follow-up CT results after 3 months (Fig 2)

In the 100 cases included in the study, the mean global CT score was 6.60 ± 8.28. The mean CT score of each lobe was as follows; 1.09 ± 1.78 for right upper lobe (RUL), 1.18 ± 1.95 for RML, 1.67 ± 2.06 for right lower lobe (RLL), 1.22 ± 1.83 for left upper lobe (LUL), and 1.44 ± 2.09 for left lower lung (LLL). 13 patients did not show any parenchymal involvement at CT and was therefore scored as 0 (Table 2).

**Fig 2:**
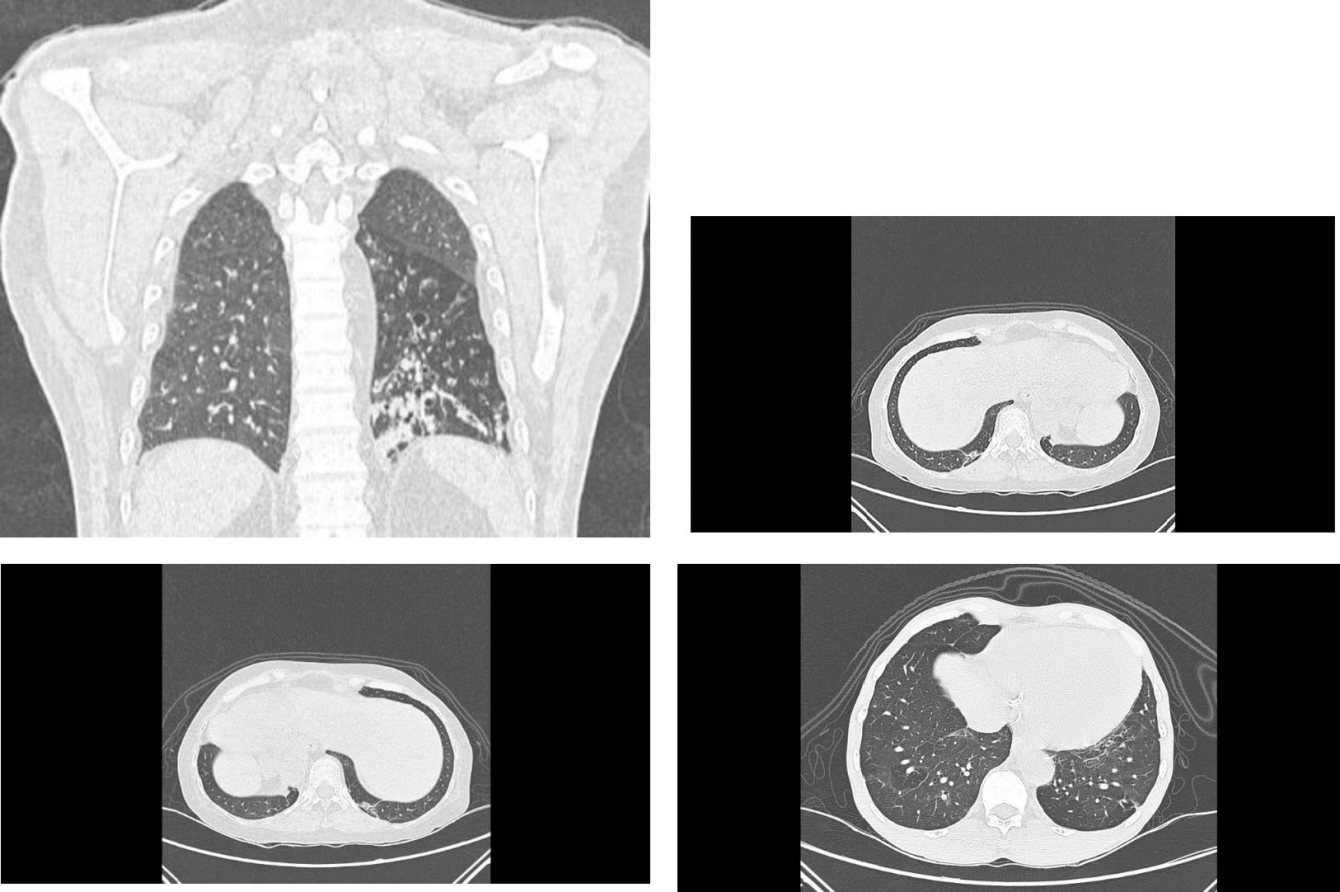
Radiological changes after COVID-19. A: Coronal (A) and axial (B) computed tomography (CT) in lung window showing evidence of bronchiectasis at basal segment of left lower lobe. B: Axial computed tomography (CT) in lung window showing evidence of ground-glass opacity at posterior aspect of both lower lungs. C: Axial computed tomography (CT) in lung window showing band-like parenchymal opacity at posterior aspect of right basal lung. D: Axial computed tomography (CT) in lung window showing reticulation at both basal lung.

**Table 2:** CT scores for each lung segment.

	n	Mean ± SD	No. of involved segments
RUL	100	1.09 ± 1.78	0.68±0.99
RML	100	1.18 ± 1.95	0.60±0.93
RLL	100	1.67 ± 2.06	0.93±1.04
LUL	100	1.22 ± 1.83	0.71±0.96
LLL	100	1.44 ± 2.09	0.82±1.04
RLL+RML+RLL	100	3.94 ± 4.96	2.21±2.42
LUL+LLL	100	2.66 ± 3.65	1.53±1.78
RLL+RML+RLL+LUL+LLL	100	6.60 ± 8.28	3.74±3.91

LLL, left lower lung; LUL, left upper lung; RLL, right lower lung; RML, right middle lung; RUL, right upper lung; SD, standard deviation.

The most common disease pattern was reticulation with the mean CT score of 2.05 ± 2.52, observed in 64 patients, followed by ground-glass opacity which had a mean CT score of 1.73 ± 4.4 observed in 32 patients. Mean CT score of bronchiectasis was 1.57 ± 4.43, found in 28 patients and mean CT score of parenchymal band was 1.25 ± 2.39, found in 43 patients. No honeycombing pattern was found and mean CT score was 0 ± 0.

### Association between various parameters and CT findings

The CT score showed no association with PFT findings for determining pulmonary function, except for the presence of bronchiectasis (Table 3). To study the factors correlated with abnormal CT findings, we extracted data regarding the number of abnormal CT images. The magnitude of abnormal CT findings did not differ in relation to the abnormal laboratory findings (white blood cell count and D-dimer and CRP levels), the mode of renal replacement therapy, and need for mechanical ventilation except for oxygen support and degree of abnormalities in PFTs (Table 4). Patients with abnormal CT findings (bronchiectasis, reticulation, and ground-glass opacities) had significantly higher oxygen requirements than those with normal CT findings (p = 0.008 for bronchiectasis and p = 0.041 for reticulation and p = 0.032 for ground-glass opacities).

**Table 3:** Comparison between radiographic findings and pulmonary function test results.

	Normal (n = 31)	Obstruction (n = 7)	Restriction (n = 41)	Small airway disease (n = 5)	Cannot interpret (n = 16)	p-value
Total score	Mean ± SD	Mean ± SD	Mean ± SD	Mean ± SD	Mean ± SD	
Bronchiectasis	0.57 ± 1.50	0.67 ± 1.21	0.95 ± 2.70	0	5.75 ± 9.01	0.001
Honeycombing	0 ± 0	0 ± 0	0 ± 0	0	0	1.000
Parenchymal band	1.11 ± 2.03	0.83 ± 0.83	1.51 ± 3.00	0	1.00 ± 1.59	0.842
Reticulation	2.17 ± 2.51	1.83 ± 1.33	1.67 ± 2.40	0	2.88 ± 3.12	0.332
Ground-glass opacity	0.97± 2.04	4.00 ± 9.80	2.14 ± 5.07	0	1.44 ± 3.20	0.827

Kruskal–Wallis H test; Significant if p < 0.05.

**Table 4:** Factors associated with abnormal CT findings.

	Normal (n = 13)		1 (n = 42)		2 (n = 21)		3+ (n = 24)		p-value
	n	% | Median (IQR)	n	% | Median (IQR)	n	% | Median (IQR)	n	% | Median (IQR)	
**WBC (10** ^**9**^ **/L)**	13	6.9 (5.18–8.17)	42	7 (4.85–9.2)	20	6.23 (5.56–9.55)	22	6.1 (4.86–7.5)	0.610
**CRP (mg/L)**	8	9.25 (3.6–28.2)	29	5.6 (3.78–30.4)	11	13.1 (4.25–48.8)	15	18 (6–83.9)	0.195
**IL-6 (pg/mL)**	0	-	7	24.2 (9.1–28.3)	2	4.54 (1.92–7.15)	3	42.5 (17.4–43.3)	0.057
**D-dimer (mg/L)**	4	1.24 (0.81–1.57)	6	1.38 (0.6–2.78)	4	0.73 (0.67–1.7)	7	1.11 (1.08–1.35)	0.917
**CKD_DM**									0.145
N	11	84.62	26	61.90	13	61.90	11	45.83	
Y	2	15.38	16	38.10	8	38.10	13	54.17	
**O**_**2**_ **support**									0.018
N	13	100	37	88.1	16	76.19	15	62.5	
Y	0	-	5	11.9	5	23.81	9	37.5	
**FVC % predicted**	13	84 (77–100)	42	78 (67–89)	20	80.5 (69–92)	23	67 (62–78)	0.020
**FEV1% predicted**	13	83 (78–112)	42	76.5 (63–88)	20	82 (69.5–94.5)	23	66 (56–79)	0.003
**FEV1 >80**									0.007
≤ 80	5	38.46	28	66.67	9	45.00	20	86.96	
>80	8	61.54	14	33.33	11	55.00	3	13.04	
**FEV1/FVC**	13	0.85 (0.8–0.88)	42	0.82 (0.79–0.88)	20	0.82 (0.77–0.87)	23	0.82 (0.76–0.86)	0.454
**FEF25-75%**	13	104 (58–140)	42	68 (53–91)	20	80.5 (47.5–95.5)	23	61 (37–93)	0.122
**FVC % change**	13	0 (-1–3.52)	42	2 (-1.74–7)	20	1 (-1–5.73)	23	0 (-8.37–4.6)	0.495
**FEV1% change**	13	4 (1.58–5.31)	42	3 (0–8)	20	3.31 (-1.06–9.62)	23	4.65 (0–9.69)	0.959

Chi-square test or Fisher’s exact test.

Kruskal-Wallis H test.

Significant if p < 0.05.

CKD, chronic kidney disease; CRP, C-reactive protein; DM, diabetes mellitus; FEV1, forced expiratory volume in 1 second (forced expiratory flow at 25%– 75% of FVC); FVC, forced vital capacity; WBC, white blood cells.

Our cohort showed significantly lower lung volume (FVC) in patients presenting three or more abnormalities in CT findings (median [IQR] FVC: 67% [62%-78%]) compared to those with normal CT findings (84% [77%-100%]), patients with one abnormal CT finding (78% [67%-89%]), and patients with two abnormal findings (80.5% [69%-92%]; *p* = 0.020%). Predicted FEV1 and FEV1/FVC (FEV1% < 80%, FEV1/FVC > 70%) indicative of restrictive ventilation dysfunction were found predominantly in those who had CT scores of 3 or more than in those who had normal CT scores or had one abnormal finding (P = 0.003 and P = 0.007, respectively).

Twenty-one patients had severe disease and 79 had non-severe disease. Various parameters were compared between the two groups (Table 5). Among the 21 patients with severe disease, eight (38.10%) showed normal PFT findings, three (14.29%) showed obstructive dysfunction, three (14.29%) showed restrictive dysfunction, and seven had data classified as “unable to interpret.” Among the 79 patients with non-severe disease, 27 (34.18%) had normal PFT findings, three (3.38%) had obstructive dysfunction, 40 (50.63%) had restrictive dysfunction, nine (11.39%) were classified as “unable to interpret” and none had small airway disease. While lung function injury was more commonly seen in severe disease, restrictive dysfunction remained the most typical finding and presented even in non-severe cases.

**Table 5:** Comparison of pulmonary function test between severe and non- severe group at follow-up.

	non severe (n = 79)		severe (n = 21)		p-value
	n	% | Median (IQR)	n	% | Median (IQR)	
**Pulmonary function test**					0.020 ^a^
Normal	27	34.18	8	38.10	
Obstructive	3	3.80	3	14.29	
Restrictive	40	50.63	3	14.29	
Small airway disease	0	0	0	-	
Cannot interpret	9	11.39	7	33.33	
**Bronchiectasis_total**	79	0 (0–0)	21	0 (0–4)	0.015^b^
**Honeycombing_total**	79	0 (0–0)	21	0 (0–0)	1.000^b^
**Parenchymal band_total**	79	0 (0–1)	21	1 (0–3)	0.035^b^
**Reticulation_total**	79	1 (0–2)	21	2 (1–6)	0.004^b^
**Ground-glass opacity_total**	79	0 (0–1)	21	0 (0–3)	0.066^b^
**WBC(10** ^**9**^ **/L)**	76	6.65 (5.1–8.8)	21	5.83 (5.3–9.06)	0.937^b^
**CRP(mg/L)**	46	7.39 (4–30.4)	17	12.7 (10.7–61.5)	0.045^b^
**IL-6 (pg/mL)**	7	24.2 (7.53–28.3)	5	21.6 (17.4–30.4)	0.935^b^
**CT score (RLL+RML+RLL+LUL+LLL)**	79	2 (1–6)	21	8 (3–24)	<0.001^b^

a exact test.

B Mann-Whitney U test.

Significant if p < 0.05.

CRP, C-reactive protein; CT, computed tomography; IL-6, interleukin-6; LLL, left lower lung; LUL, left upper lung; RLL, right lower lung; RML, right middle lung; RUL, right upper lung; WBC, white blood count.

CT image characteristics were evaluated and compared between severe and non-severe disease. Of the 21 cases classified as showing severe COVID-19 in the study, the median CT score was 8 [IQR, 3–24], in-line with the findings of the previous study [17]. We found that the scores for bronchiectasis, parenchymal bands, and reticulation in the severe group were significantly higher than those in the non-severe group (p = 0.015, 0.035, and 0.004, respectively). The CRP level was significantly higher in the severe than the non-severe group (median, 12.4 mg/L [10.7–61.5 mg/L]) while white blood counts and IL-6 were not significantly different between the two groups.

### Symptoms after COVID-19 infection (Fig 3, S1, S2 and S3 Tables in S2 File)

The respiratory symptoms at three months or more post COVID-19 recovery included persistent cough alone in six patients, dyspnea alone in 14 patients, and concomitant cough and dyspnea in four patients. The patients with and without respiratory symptoms at follow-up showed no significant differences in PFT results, CT scan findings, and laboratory data.

**Fig 3:**
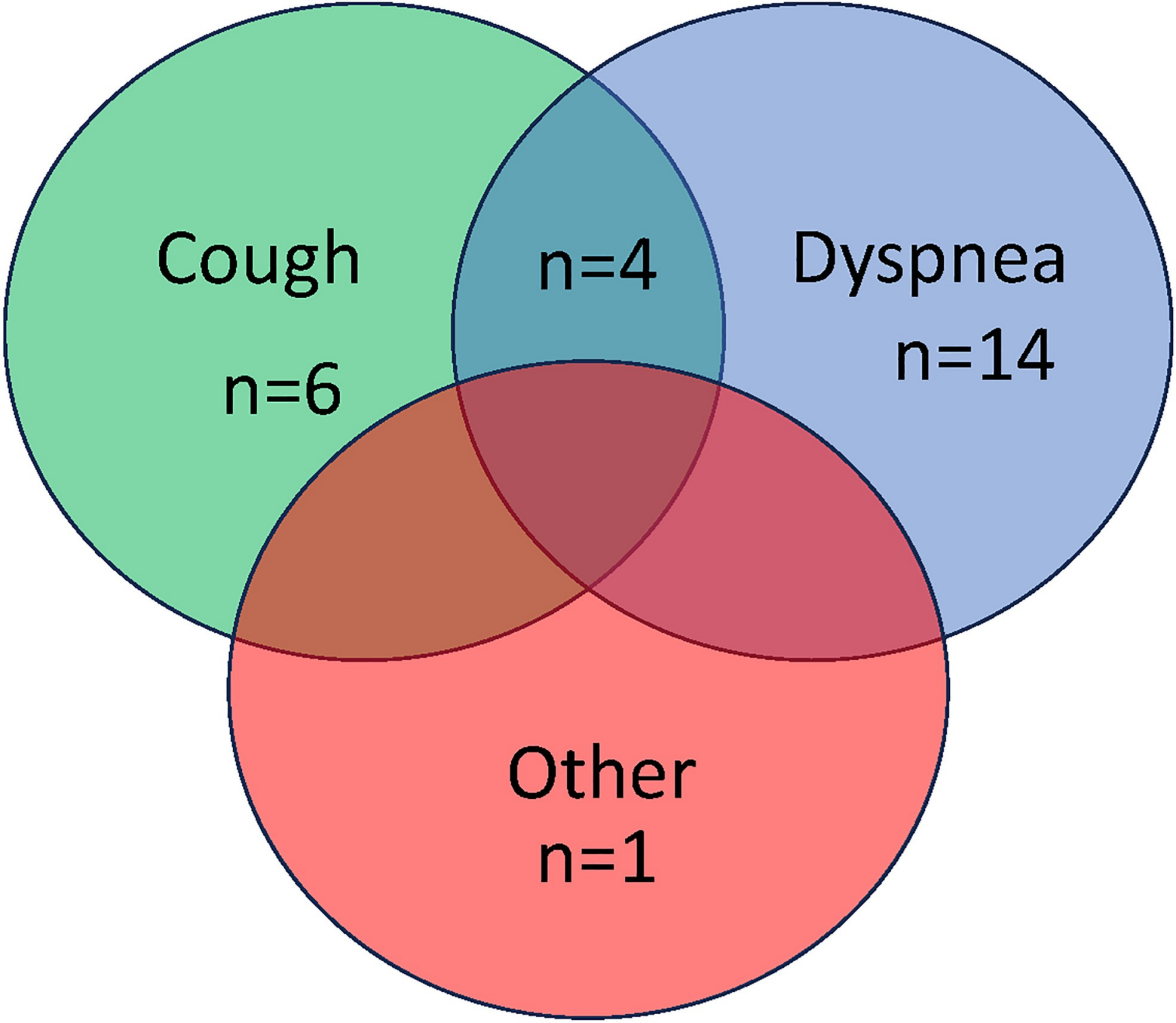
Symptoms after COVID-19 infection. Out of 100 patients, 21 patients had one or more ongoing respiratory symptoms (dyspnea, cough, or other). Six patients had persistent cough, 14 patients had dyspnea, four had both cough and dyspnea,and one patient had other symptoms.

## Discussion

Differences in the prevalence and severity of pulmonary disease after COVID-19 infection, as determined by pulmonary function testing and chest CT, in CKD and healthy population remains to be determined. To our knowledge, this is the first study reporting the respiratory follow-up data in CKD patients after SARS-CoV-2 infection. A total of 100 patients with an average recovery time of 4 months were included in the study. Our data suggested that restrictive lung diseases and bronchiectasis are the most frequent lung abnormalities in this population. Furthermore, these two abnormalities were significantly associated with each other, potentially indicating evolving pulmonary fibrosis after COVID-19 [19–21]. One study also showed a high prevalence of pulmonary fibrosis, as determined by chest CT, after COVID-19 infection [22], and another SARS-CoV-1 study reported abnormal chest radiography results in 30% of survivors at the 6-month follow-up [23]. Acute COVID-19 typically presents with ground-glass opacities, alveolar consolidations, parenchymal bands, and imaging findings indicating organizing pneumonia [24]. The most frequent radiologic abnormalities observed at the short-term follow-up typically include ground-glass opacities, consolidation, and parenchymal bands [25]. Our cohort was limited to only CKD patients, which is a very specific population. Thus, our patients may have had unknown preexisting comorbid diseases, despite our efforts to exclude patients with chronic lung disease before enrollment. Underlying CKD may also contribute to lung congestion that shows a hazy appearance on chest CT. Future studies should address whether CT lesions will improve or persist and whether any residual radiographic sequelae will have long-term clinical and functional significance.

Overall, one-third of our patients showed normal PFT results. Patients with severe COVID-19 infection still had normal PFT results, and only one patient showed severe obstructive disease. The remaining patients in the severe group had normal PFT results with mild abnormalities, while those in the non-severe group showed residual restrictive impairment at most. Previous studies have shown low-normal to significantly decreased lung volumes (TLC, FVC and FEV1) in patients after severe COVID-19 [26]. They also demonstrated a negative correlation between the duration of mechanical ventilation during the acute disease phase and pulmonary function at the 4-month follow-up. Requirement of oxygen (but not mechanical ventilation) during illness denoted subsequent pulmonary function in our study.

Lung function abnormalities after COVID-19 may depend on several other factors. Bottino et al followed 16 asymptomatic and mildly symptomatic pediatric patients with COVID-19 for ≥30 days and found that none of them showed abnormal results for spirometry, lung ultrasound, or diffusing capacity for carbon monoxide (DLCO) [27]. Additionally, Verzi et al [28]. reported the findings for 75 pediatric patients 1–3 months after infection and found only a minority of patients having a pulmonary function sequelae, and the numbers were similar in both asymptomatic and severe groups. However, studies in adult COVID-19 patients showed the prevalence of restrictive pulmonary dysfunction to be 44.3%, with impaired DLCO being the most frequently observed finding in 34.8% of the patients (95% CI, 25.8–43.8; *I*^*2*^ = 91.5%) [29]. Thus, age may be an important factor affecting long-term results [30]. Data on pulmonary function after COVID-19 in dialysis patients are scarce, and the dialysis population shows several risk factors for severe manifestations of COVID-19, including advanced age and multiple underlying comorbid conditions. Despite their heightened risk, the pulmonary abnormalities in the population of this present study aligns with the findings of the normal population present in the above mentioned previous studies. The other explanation could be patient selection as the population included in the study were mild to moderate cases having good prognosis.

As the severity of lung involvement progressed from one to three or more abnormal radiographic findings, PFT results also deteriorated. The radiological and functional abnormalities observed in our study may represent residual damage after COVID-19 alone or in addition to pre-existing lung abnormalities caused by CKD, indicating the need for long-term follow-up. Although follow-up assessments showed some patients with residual symptoms, these symptoms did not correlate with any of our measured parameters. In the study conducted by Moreno-Pe’rez et al. [31], the authors described no association between the perceived symptoms and abnormal spirometry and chest radiograph findings. However, neither their study nor the present study included diffusing capacity as a measurement. Laboratory findings were also not associated with any symptoms. Only the CRP level was associated with disease severity, and it could be a useful marker of severity during acute illness.

This study yielded numerous important findings. Data regarding pulmonary sequelae after COVID-19 in the dialysis population are very limited, and our study was the first to report such findings. Additionally, we conducted both imaging and lung-function assessments simultaneously.

The primary limitation of the study was the lack of baseline PFT and CT data prior to COVID-19, since it was not possible to predict who will contract the disease. Consequently, the PFT and CT abnormalities could not be presumed to be directly attributable to the COVID-19 infection. The data from previous reports showed that restrictive lung disease was highly prevalent in HD and continuous peritoneal ambulatory peritoneal dialysis (CAPD) patients, consistent with pulmonary venous congestion [32] However, we excluded patients with known prior pulmonary disease, so the enrolled patients can be assumed to have had normal PFT data prior to the COVID-19 infection. Second, our patients were frequently in a volume-overloaded state due to the dialysis procedure, and this may have interfered with CT results, especially in patients showing ground-glass appearance on CT. Therefore, we chose to perform the CT scans on post-dialysis day to maintain the dry weight of the patient as much as possible. Third, we did not perform the DLCO testing, which is the most sensitive test to detect early airway dysfunction. Finally, the study population included too few patients undergoing peritoneal dialysis (PD), limiting the assessment of statistical differences between dialysis modalities.

## Conclusion

Our study yielded important insights into persistent respiratory symptoms and impaired pulmonary function, as evidenced by CT lung abnormalities and pulmonary function testing, in CKD stage 5 patients undergoing dialysis. The overall abnormalities were not different from those in the normal population with COVID-19 as assessed in previous studies. The most common pattern observed on PFT was restrictive dysfunction, and the most common CT finding was the presence of reticulations. Only 10% of patients showed persistent respiratory symptoms that did not correlate with CT or PFT results. Longitudinal follow-up and repeat PFT and CT scans in a larger dialysis population, including patients receiving CAPD, are needed to better understand the pulmonary sequelae in this unique population.

## Supporting information

S1 Checklist (DOCX)

S1 File (DOCX)

S2 File S1-S3 Table: Comparison between Long COVID symptoms with follow up pulmonary function test. (DOCX)

S1 Appendix SpiroThai for calculate the standard value of lower limit of normal by online by web browser or mobile device. (DOCX)
