# Supplementary material for: Follow-up evaluation of pulmonary function and computed tomography findings in chronic kidney disease patients after COVID-19 infection
Source: PLoS One. 2023 Aug 15;18(8):e0286832. doi: 10.1371/journal.pone.0286832 (PMC10427007; doi:10.1371/journal.pone.0286832)
Supplement: S1 File — (DOCX) [file pone.0286832.s002.docx]

**Case record form**

**Follow-up Study of the Pulmonary Function and CT scan finding in Chronic Kidney Disease Patients After COVID-19 Infection**

No ………………….

Enrolled date ………………….

| **Criteria** |  | |
| --- | --- | --- |
| **Inclusion criteria (Must have all items)** | **Yes** | **No** |
| Age 18-80 year |  |  |
| CKD stage 5 on kidney replacement therapy either HD or CAPD for more than 3 months |  |  |
| Has been diagnosed with COVID-19 and recovered more than 3 months |  |  |
| **Exclusion criteria** | **Yes** | **No** |
| - Underlying chronic lung diseases (COPD, significant fibrosis) |  |  |

| **Baseline characteristic** | | | |
| --- | --- | --- | --- |
| **Sex** | 🞏 Male 🞏 Female | **Age** | ____ Year |
| **Body weight** | _____ Kg. | **Height** | _____ cm. |
| **CKD etiology** | 🞏 DM 🞏 HT 🞏 Glomerular disease  🞏 Obstructive uropathy 🞏 Others 🞏 Unknown | | |
| **Renal replacement therapy** | 🞏 Hemodialysis | 🞏 Peritoneal dialysis |  |
| **Dialysis vintage** | _____ Year | | |
| **Post-covid (from diagnosis to recruit to CT)** | ______ Month | **Admission day** | ____ Day |
| **Admission** | 🞏 MICU/RCU/CCU  (at least one transfer to ICU or on respirator) | 🞏 Isolation ward | 🞏 hospitel |
| **Co-morbid disease** | | | |
| **Diabetes mellitus** | 🞏 Yes | 🞏 No |  |
| **Hypertension** | 🞏 Yes | 🞏 No |  |
| **Ischemic heart disease** | 🞏 Yes | 🞏 No |  |
| **Heart failure with LVEF < 45** | 🞏 Yes | 🞏 No |  |
| **Atrial fibrillation** | 🞏 Yes | 🞏 No |  |
| **HIV** | 🞏 Yes | 🞏 No |  |
| **Cancer** | 🞏 Yes | 🞏 No |  |
| **Current active smoker** | 🞏 Yes | 🞏 No |  |
| **Previous medication** | | | |
| **ACEI or ARB** | 🞏 Yes | 🞏 No |  |
| **Antiplatelets** | 🞏 Yes | 🞏 No |  |
| **Oral anticoagulation** | 🞏 Yes | 🞏 No |  |
| **Immunosuppressive drug** | 🞏 Yes | 🞏 No |  |
| **Vaccination prior COVID-19 infection** | 🞏 unvaccinated 🞏 only 1 SV/SP 🞏 Only 1 AZ 🞏 only 1 mRNA  🞏 2 SV/SP 🞏 2 AZ 🞏 2 mRNA 🞏 AZ+mRNA 🞏 2SV/SP+AZ  🞏 2 SV/SP + mRNA 🞏 Other vaccine or other combination | | |
| **Treatment of COVID-19** | | | |
| **Flavipiravir** | 🞏 Yes | 🞏 No |  |
| **Andrographolide** | 🞏 Yes | 🞏 No |  |
| **Remdisivir** | 🞏 Yes | 🞏 No |  |
| **Corticosteroid** | 🞏 Yes | 🞏 No |  |
| **Tocilizumab** | 🞏 Yes | 🞏 No |  |
| **Baciritinib** | 🞏 Yes | 🞏 No |  |
| **Hemoperfusion** | 🞏 Yes | 🞏 No |  |
| **IV anticoagulation** | 🞏 Yes | 🞏 No |  |
| **Ivermectin** | 🞏 Yes | 🞏 No |  |
| **Mechanical ventilation** | 🞏 More than 7 days | 🞏 Less than 7 days | 🞏 No |
| **Oxygen support** | 🞏 More than 7 days | 🞏 Less than 7 days | 🞏 No |
| **Oxygen flow rate (L/min) at diagnosis** | ______ L/min | **Oxygen maximal flow rate (L/min)** | ________ L/min |
| **Investigation during admission (first day)** | | | |
| **Hemoglobin** | ____ g/dL | **WBC** | ______ |
| **PMN** | _____ % | **Lymph** | _____ % |
| **Eosinophil** | ______ % | **Platelet** | ________________ |
| **CRP** | ______ mg/dL | **IL-6** | _______ |
| **Alb** | ______ g/L | **Ferritin** | ______ ug/L |
| **Fibrinogen** | _______ g/L | **D-dimer** | ______ ug/mL |
| **Imaging** (Chest x-ray or CT chest at discharge) | | | |
| **Lesions** | 🞏 Bilateral | 🞏 Unilateral | 🞏 No lesion |
| **Patchy opacities** | 🞏 Bilateral | 🞏 Unilateral | 🞏 No lesion |
| **Fibrous stripes** | 🞏 Bilateral | 🞏 Unilateral | 🞏 No lesion |
| **Pleural effusion** | 🞏 Bilateral | 🞏 Unilateral | 🞏 No lesion |
| **HRCT chest at study** | | | |
| **Lesions** | 🞏 Bilateral | 🞏 Unilateral | 🞏 No lesion |
| **Intraparenchymal opacities** | 🞏 Bilateral | 🞏 Unilateral | 🞏 No lesion |
| **Ground glass opacities** | 🞏 Bilateral | 🞏 Unilateral | 🞏 No lesion |
| **Nonemphysematous cyst** | 🞏 Bilateral | 🞏 Unilateral | 🞏 No lesion |
| **Centrilobular nodules** | 🞏 Bilateral | 🞏 Unilateral | 🞏 No lesion |
| **Reticulation** | 🞏 Bilateral | 🞏 Unilateral | 🞏 No lesion |
| **Honeycombing** | 🞏 Bilateral | 🞏 Unilateral | 🞏 No lesion |
| **Traction bronchiectasis** | 🞏 Bilateral | 🞏 Unilateral | 🞏 No lesion |
| **Lung function and 6 minutes walk test** | | | |
| **Reduced FVC below LLN** | 🞏 Yes | 🞏 No |  |
| **Reduced FEV1/FVC** | 🞏 Yes | 🞏 No |  |
| **Reduced DLCO** | 🞏 Yes | 🞏 No |  |
| **Reduced 6MWT < 80 percentile of predict value** | 🞏 Yes | 🞏 No |  |
| **Cough visual score > 20** | 🞏 Yes | 🞏 No |  |
