# Supplementary material for: Follow-up evaluation of pulmonary function and computed tomography findings in chronic kidney disease patients after COVID-19 infection
Source: PLoS One. 2023 Aug 15;18(8):e0286832. doi: 10.1371/journal.pone.0286832 (PMC10427007; doi:10.1371/journal.pone.0286832)
Supplement: S2 File — (DOCX) [file pone.0286832.s003.docx]

| **Supplement Table 1 : Comparison between Long COVID symptoms with follow up pulmonary function test** | | | | |  |  |
| --- | --- | --- | --- | --- | --- | --- |
|  | no (n=94) | | **cough (n=6)** | | p-value |  |
|  | n | % \| Median (IQR) | n | % \| Median (IQR) |  |  |
| **interpretation** |  |  |  |  | 0.271 |  |
| Normal | 31 | 32.98 | 0 | - |  |  |
| Obstruction | 7 | 7.45 | 0 | - |  |  |
| restriction | 37 | 39.36 | 4 | 66.67 |  |  |
| Small airway disease | 5 | 5.32 | 0 | - |  |  |
| Cannot interprete | 14 | 14.89 | 2 | 33.33 |  |  |
| **Bronchiectasis_total** | 94 | 0 (0 - 1) | 6 | 0.5 (0 - 1) | 0.309 |  |
| **Honeycombing_total** | 94 | 0 (0 - 0) | 6 | 0 (0 - 0) | 1.000 |  |
| **Parenchymal band_total** | 94 | 0 (0 - 1) | 6 | 0 (0 - 1) | 0.583 |  |
| **Reticulation_total** | 94 | 1 (0 - 3) | 6 | 2 (0 - 2) | 0.988 |  |
| **Ground-glass opacity_total** | 94 | 0 (0 - 1) | 6 | 1 (0 - 4) | 0.289 |  |
| **WBC** | 91 | 6.36 (5.2 - 9) | 6 | 6.34 (4.86 - 8.4) | 0.863 |  |
| **CRP** | 59 | 12 (4.3 - 43.8) | 4 | 9.95 (4.76 - 15.35) | 0.563 |  |
| **IL-6** | 11 | 24.2 (7.53 - 30.4) | 1 | 17.4 | 0.664 |  |
| Fisher's exact test |  |  |  |  |  |  |
| Mann-Whitney U test |  |  |  |  |  |  |
| Significant if p<0.05  WBC,White blood cell;CRP.C-reactive protein;IL-6,interleukin-6 |  |  |  |  |  |  |
|  |  |  |  |  |  |  |
|  |  |  |  |  |  |  |
| **Supplement Table 2: Comparison between Long COVID symptoms with follow up pulmonary function test** | | | | |  |  |
|  | no (n=86) | | **dyspnea (n=14)** | | p-value |  |
|  | n | % \| Median (IQR) | n | % \| Median (IQR) |  |  |
| **interpretation** |  |  |  |  | 0.456 |  |
| Normal | 28 | 32.56 | 3 | 21.43 |  |  |
| Obstruction | 6 | 6.98 | 1 | 7.14 |  |  |
| restriction | 32 | 37.21 | 9 | 64.29 |  |  |
| Small airway disease | 5 | 5.81 | 0 | - |  |  |
| Cannot interprete | 15 | 17.44 | 1 | 7.14 |  |  |
| **Bronchiectasis_total** | 86 | 0 (0 - 1) | 14 | 0 (0 - 1) | 0.995 |  |
| **Honeycombing_total** | 86 | 0 (0 - 0) | 14 | 0 (0 - 0) | 1.000 |  |
| **Parenchymal band_total** | 86 | 0 (0 - 1) | 14 | 0 (0 - 1) | 0.982 |  |
| **Reticulation_total** | 86 | 1 (0 - 3) | 14 | 2 (2 - 3) | 0.060 |  |
| **Ground-glass opacity_total** | 86 | 0 (0 - 1) | 14 | 0 (0 - 2) | 0.796 |  |
| **WBC** | 84 | 6.34 (5.25 - 8.8) | 13 | 7.8 (4.86 - 9.4) | 0.653 |  |
| **CRP** | 53 | 12 (4.4 - 48.8) | 10 | 7.6 (3.5 - 12.7) | 0.259 |  |
| **IL-6** | 11 | 24.2 (7.53 - 30.4) | 1 | 17.4 | 0.664 |  |
| Fisher's exact test |  |  |  |  |  |  |
| Mann-Whitney U test |  |  |  |  |  |  |
| Significant if p<0.05  WBC,White blood cell;CRP.C-reactive protein;IL-6,interleukin-6 |  |  |  |  |  |  |
|  |  |  |  |  |  |  |
|  |  |  |  |  |  |  |
| **Supplement Table 3: Comparison between Long COVID symptoms with follow up pulmonary function test (PFT)** | | | | |  |  |
|  | no (n=99) | | **other (n=1)** | | p-value |  |
|  | n | % \| Median (IQR) | n | % \| Median (IQR) |  |  |
| **PFT results** |  |  |  |  | 0.999 |  |
| Normal | 31 | 31.31 | 0 | - |  |  |
| Obstruction | 7 | 7.07 | 0 | - |  |  |
| restriction | 40 | 40.4 | 1 | 100 |  |  |
| Small airway disease | 5 | 5.05 | 0 | - |  |  |
| Cannot interprete | 16 | 16.16 | 0 | - |  |  |
| **Bronchiectasis total score** | 99 | 0 (0 - 1) | 1 | 0 | NA |  |
| **Honeycombing total score** | 99 | 0 (0 - 0) | 1 | 0 | NA |  |
| **Parenchymal band total score** | 99 | 0 (0 - 1) | 1 | 0 | NA |  |
| **Reticulation total score** | 99 | 1 (0 - 3) | 1 | 1 | NA |  |
| **Ground-glass opacity total score** | 99 | 0 (0 - 1) | 1 | 0 | NA |  |
| **WBC** | 96 | 6.38 (5.19 - 8.95) | 1 | 5.6 | NA |  |
| **CRP** | 63 | 12 (4.3 - 34.4) | 0 | - | NA |  |
| **IL-6** | 12 | 22.9 (8.32 - 29.35) | 0 | - | NA |  |
| Fisher's exact test |  |  |  |  |  |  |
| Mann-Whitney U test |  |  |  |  |  |  |
| Significant if p<0.05  WBC,White blood cell;CRP.C-reactive protein;IL-6,interleukin-6 |  |  |  |  |  |  |
|  |  |  |  |  |  |  |
|  |  |  |  |  |  |  |
