# Supplementary material for: Follow-up evaluation of pulmonary function and computed tomography findings in chronic kidney disease patients after COVID-19 infection
Source: PLoS One. 2023 Aug 15;18(8):e0286832. doi: 10.1371/journal.pone.0286832 (PMC10427007; doi:10.1371/journal.pone.0286832)

| **S1 Table: Comparison between Long COVID symptoms with FU pulmonary function test** | | | | |  |
| --- | --- | --- | --- | --- | --- |
|  | no (n=94) | | **res.cough (n=6)** | | p-value |
|  | n | % \| Median (IQR) | n | % \| Median (IQR) |  |
| **interpretation** |  |  |  |  | 0.102^a^ |
| Normal | 35 | 37.23 | 0 | - |  |
| Obstruction | 5 | 5.32 | 1 | 16.67 |  |
| restriction | 40 | 42.55 | 3 | 50.00 |  |
| Small airway disease | 0 | - | 0 | - |  |
| Cannot interprete | 14 | 14.89 | 2 | 33.33 |  |
| **Bronchiectasis_total** | 94 | 0 (0 - 1) | 6 | 0.5 (0 - 1) | 0.309 ^b^ |
| **Honeycombing_total** | 94 | 0 (0 - 0) | 6 | 0 (0 - 0) | 1.000 ^b^ |
| **Parenchymal band_total** | 94 | 0 (0 - 1) | 6 | 0 (0 - 1) | 0.583 ^b^ |
| **Reticulation_total** | 94 | 1 (0 - 3) | 6 | 2 (0 - 2) | 0.988 ^b^ |
| **Ground-glass opacity_total** | 94 | 0 (0 - 1) | 6 | 1 (0 - 4) | 0.289 ^b^ |
| **WBC (10^9^/L)** | 91 | 6.36 (5.2 - 9) | 6 | 6.34 (4.86 - 8.4) | 0.863 ^b^ |
| **CRP (mg/L)** | 59 | 12 (4.3 - 43.8) | 4 | 9.95 (4.76 - 15.35) | 0.563 ^b^ |
| **IL-6 (pg/mL)** | 11 | 24.2 (7.53 - 30.4) | 1 | 17.4 | NA |
| ^a^ Fisher's exact test |  |  |  |  |  |
| ^b^ Mann-Whitney U test |  |  |  |  |  |
| Significant if p<0.05 |  |  |  |  |  |

| **S2 Table: Comparison between Long COVID symptoms with follow up pulmonary function test** | | | | |  |
| --- | --- | --- | --- | --- | --- |
|  | no (n=86) | | **dyspnea (n=14)** | | p-value |
|  | n | % \| Median (IQR) | n | % \| Median (IQR) |  |
| **interpretation** |  |  |  |  | 0.374 ^a^ |
| Normal | 31 | 36.05 | 4 | 28.57 |  |
| Obstruction | 4 | 4.65 | 2 | 14.29 |  |
| restriction | 36 | 41.86 | 7 | 50.00 |  |
| Small airway disease | 0 | - | 0 | - |  |
| Cannot interprete | 15 | 17.44 | 1 | 7.14 |  |
| **Bronchiectasis_total** | 86 | 0 (0 - 1) | 14 | 0 (0 - 1) | 0.995 ^b^ |
| **Honeycombing_total** | 86 | 0 (0 - 0) | 14 | 0 (0 - 0) | 1.000 ^b^ |
| **Parenchymal band_total** | 86 | 0 (0 - 1) | 14 | 0 (0 - 1) | 0.982 ^b^ |
| **Reticulation_total** | 86 | 1 (0 - 3) | 14 | 2 (2 - 3) | 0.060 ^b^ |
| **Ground-glass opacity_total** | 86 | 0 (0 - 1) | 14 | 0 (0 - 2) | 0.796 ^b^ |
| **WBC(10^9^/L)** | 84 | 6.34 (5.25 - 8.8) | 13 | 7.8 (4.86 - 9.4) | 0.653 ^b^ |
| **CRP (mg/L)** | 53 | 12 (4.4 - 48.8) | 10 | 7.6 (3.5 - 12.7) | 0.259 ^b^ |
| **IL-6(pg/mL)** | 11 | 24.2 (7.53 - 30.4) | 1 | 17.4 | NA |
| ^a^ Fisher's exact test |  |  |  |  |  |
| ^b^ Mann-Whitney U test |  |  |  |  |  |
| Significant if p<0.05 |  |  |  |  |  |

| **S 3 Table: Comparison between Long COVID symptoms with FU pulmonary function test** | | | | | |  |  |
| --- | --- | --- | --- | --- | --- | --- | --- |
|  | no (n=99) | **Other (n=1)** | | | | p-value | |
|  | n | | % \| Median (IQR) | n | % \| Median (IQR) |  | |
| **interpretation** |  | |  |  |  | 0.999 ^a^ | |
| Normal | 35 | | 35.35 | 0 | - |  | |
| Obstruction | 6 | | 6.06 | 0 | - |  | |
| restriction | 42 | | 42.42 | 1 | 100.00 |  | |
| Small airway disease | 0 | | - | 0 | - |  | |
| Cannot interprete | 16 | | 16.16 | 0 | - |  | |
| **Bronchiectasis_total** | 99 | | 0 (0 - 1) | 1 | 0 | NA | |
| **Honeycombing_total** | 99 | | 0 (0 - 0) | 1 | 0 | NA | |
| **Parenchymal band_total** | 99 | | 0 (0 - 1) | 1 | 0 | NA | |
| **Reticulation_total** | 99 | | 1 (0 - 3) | 1 | 1 | NA | |
| **Ground-glass opacity_total** | 99 | | 0 (0 - 1) | 1 | 0 | NA | |
| **WBC(10^9^/L)** | 96 | | 6.38 (5.19 - 8.95) | 1 | 5.6 | NA | |
| **CRP(mg/L)** | 63 | | 12 (4.3 - 34.4) | 0 | - | NA | |
| **IL-6(pg/mL)** | 12 | | 22.9 (8.32 - 29.35) | 0 | - | NA | |
| ^a^ Fisher's exact test |  | |  |  |  |  | |
| Significant if p<0.05 |  | |  |  |  |  | |

**Supplementary Appendix 1**

**Copyright**

[Download SpiroThai 3.0](https://thaichest.files.wordpress.com/2019/08/spirothai3.zip) for the database Spirometry
[Download SpiroThai 4.0](https://thaichest.files.wordpress.com/2021/12/spirothai4.zip) for the database Complete pulmonary function test

**GLI-2012 for Thai Dashboard**

For calculate the standard value of lower limit of normal by online by web browser or mobile device
[GLI2012forThai](https://wdviz.shinyapps.io/SpiroThaiDashBoard/)


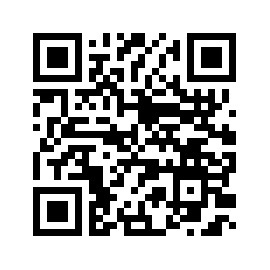

Supplement: S1 Appendix — (DOCX) [file pone.0286832.s004.docx]
